# Impact dynamics of granular debris flows based on a small-scale physical model

**DOI:** 10.1007/s11440-023-02116-8

**Published:** 2023-11-21

**Authors:** Christian Scheidl, Caroline Friedl, Lukas Reider, Susanna Wernhart, Anna-Lisa Fuchs, Anna Lisa Dankwerth, Georg Nagl, Roland Kaitna, Dirk Proske

**Affiliations:** 1 Institute of Mountain Risk Engineering (IAN), University of Natural Resources and Life Sciences, Peter-Jordan-Strasse 82, 1190 Vienna, Austria; 2 School of Architecture, Wood and Civil Engineering, Fachbereich Bauingenieurwesen, Berner Fachhochschule, Pestalozzistrasse 20, 3401 Burgdorf, Bern, Switzerland

**Keywords:** Bulk density at impact, Debris-flow impact, Physical modelling, Run-up height, Stress anisotropy

## Abstract

The peak pressure of a granular debris flow at low Froude conditions can be calculated with knowledge of the stress anisotropy and the bulk density as well as the run-up height at impact. Based on a small-scale physical model, measurements of stress anisotropy and flow density values at impact are presented and applied to existing run-up prediction models, and further compared with back-calculated run-up coefficients from measured maximum impact pressures. For this purpose, we conducted 17 experiments with impact measurements and six experiments without impact measurements at Froude numbers, ranging from 0.84 to 2.41. Our results indicate that run-up heights are best reproduced by predictive models, either based on energy or mass and moment conservation, when anisotropic stress conditions, found in this study to range from 1.2 to 5.0, and bulk density variations due to impact, ranging in this study from 0.8 to 2.3, are considered. The influence of stress anisotropy and density variation on the run-up prediction differs, depending on the modelling approach. For the calculation of run-up heights based on the energy conservation concept, the influence of stress anisotropy becomes more significant with increasing Froude number, whereas for models based on mass and momentum conservation, bulk density variations have a greater influence on the estimation of the potential run-up.

## Introduction

**Fig. 1:**
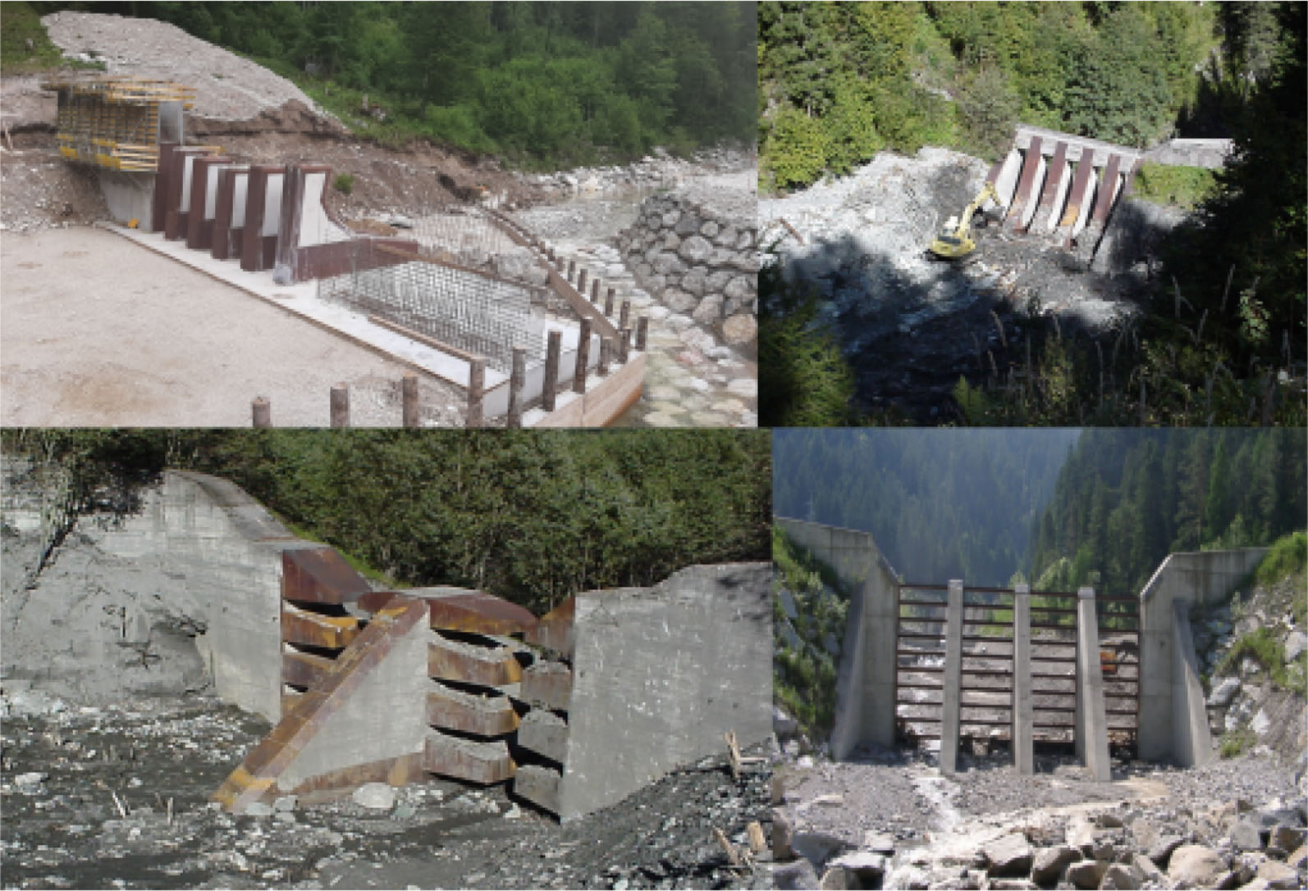
Active debris-flow specific mitigation measurements in torrents. Above from left to right: Rindbach (Upper Austria-AT); Luggauerbach (Salzburg-AT). Below from left to right: Luggauerbach (Salzburg-AT); Naisbach (South Tyrol-IT)

Debris flows are among the most damaging natural hazard processes in mountainous regions and expected climate change as well as an increasing settlement on exposed areas make specific protective mitigation structures and their maintenance increasingly important [29, 47]. Such processes can relocate a considerable quantity of sediments from steep headwater catchments into populated areas [43, 50]. Debris-flow impact analysis is therefore a key element of engineering design (Fig. 1) and risk assessment [2, 15, 16, 19], and there has been a steady increase in the number of studies on modelling impact effects of debris flows in recent years [5, 12, 15, 32, 37, 39, 49].

From an engineering prospective, conventional and widely used approaches to estimate peak impact pressures of debris flows on a vertical obstacle assume that the occurring forces are either proportional to hydrostatic or hydrodynamic pressure conditions [1, 2, 34, 38]. Here, proportionality is based on empirical evidence, accounting for the non-Newtonian flow behaviour of debris flows. However, because of the large number of different flow mixtures and the associated development of residual stresses that determine deformation and impact, the documented proportionality ratios, which are often back-calculated either from laboratory or field measurements, extend over a wide range [6, 7, 10, 15, 44, e.g.]. The greatest uncertainties exist for granular debris flows with slow impact dynamics, i.e. low Froude numbers. However, such debris-flow processes are mainly reported from field observations [9, 31, 55]. The applicability of empirical impact models thus seem to be limited especially for debris flows consisting of granular material that has an internal strength due to its frictional or collisional properties [10, 12, 46].

Faug [13] proposed a simple analytic model to estimate the impact force of a granular flow on a wall. His model is based on the assumption that the mechanical energy of the incoming flow (sum of kinetic energy and potential energy) is transformed into potential energy without any major loss of energy when the flow velocity at impact gets zero:

1 $$\begin{aligned} \frac{1}{2}\rho _0 v_0^{2} + \kappa _0\rho _0gh_0\cos \theta = \kappa _1\rho _1gh_1\cos \theta \end{aligned}$$

**Fig. 2:**
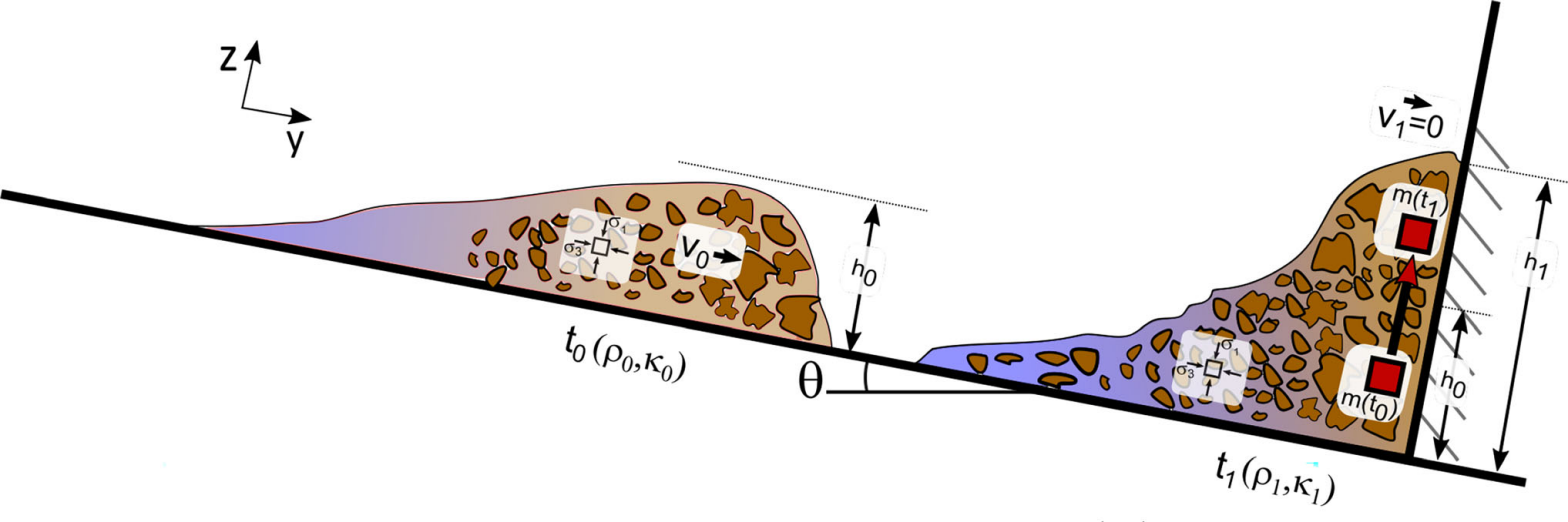
Schematic illustration of a debris-flow approaching ($t_0$) and at the moment of impact on a vertical wall ($t_1$), following energy conversion of a point mass over time $m(t_0)\rightarrow m(t_1)$ along the vertical barrier

In Eq. (1), $\rho$, *v* and *h* refer to the bulk density, flow velocity and flow height of the debris flow, with the subscript 0 denoting incoming flow conditions and subscript 1 referring to the moment at impact (c.f.: Fig. 2).

The parameter $\kappa$ reflects normal stress anisotropy and is often referred to Rankine’s earth pressure theory—given as a proportional ratio between the bed-lateral stress $\sigma _3$ and bed-normal stress $\sigma _1$ [17, 20, 22, 36, 42, 45, 46].

2 $$\begin{aligned} \kappa =\frac{\sigma _3}{\sigma _1} \end{aligned}$$

With $\kappa =1$, the acting forces correspond to the weight of the fluid in a dynamic state, $\kappa <1$ represents an active expansion of the moving masses occurs, whereas $\kappa >1$ describes a passive compression. For debris flows, typical values of $\kappa$ have been reported to range from 0.2 to 5.0 [17, 25].

The assumption of energy conservation implies that a gradual build-up of the bulk mixture takes place on impact and should thus be valid for low Froude numbers (Fig. 2). The total impact force *F*, acting on the wall with a width *w*, is then calculated as:

3 $$\begin{aligned} F = \int _{0}^{h_1} \kappa _1\rho _1g(h_1-z)w\cos \theta \,dz = \frac{1}{2}\kappa _1\rho _1gh_1^2w\cos \theta \end{aligned}$$

The impact model (Eq. 3), written in terms of a total force distributed over unit area, thus yields to the peak pressure averaged over the run-up height $h_1$:

4 $$\begin{aligned} p_{{\rm peak}} = \kappa _1\rho _1gh_1\cos \theta \end{aligned}$$

Note that Eq. (4) is only valid at low Froude numbers when limited energy dissipation can be expected. By introducing a dimensionless run-up coefficient at impact,

5 $$\begin{aligned} \beta = \frac{h_1}{h_0} \end{aligned}$$

the peak pressure exerted by debris flows can then be related to the incoming flow height ($h_0$) with:

6 $$\begin{aligned} p_{{\rm peak}} = \kappa _1\rho _1g\beta h_0\cos \theta \end{aligned}$$

Equation (6) is of great practical importance as it relates the maximum impact to the run-up height, which in turn is a critical measure for the design of protective structures or for determining critical overtopping heights. The consideration of run-up height to approximate a possible maximum impact pressure further allows the use of post-event geomorphological field data, such as flow marks on trees, rocks or walls.

According to [13], the dimensionless run-up coefficient $\beta$ with respect to energy conservation for slower debris flows (Eq. 1) can be estimated by:

7 $$\begin{aligned} \beta =\frac{1}{\kappa _0}\frac{\rho _0}{\rho _1}\left( \kappa _1+\frac{{\rm Fr}^2}{2}\right) \end{aligned}$$

with ${\rm Fr}$ the Froude number, defined as:

8 $$\begin{aligned} {\rm Fr}=\frac{v_0}{\sqrt{gh_0\cos \theta }} \end{aligned}$$

Equation (7) accounts for stress anisotropy as well as flow density variations between incoming and impacted bulk flow. Because of the sudden compression on impact, it can be assumed that $\kappa _1$ is greater than $\kappa _0$ and in any case greater than unity. If we assume the normal stresses of the incoming flow to be isotropic ($\kappa _0=1$,) almost uniform and in a steady state, a conservative estimation of the dimensional run-up-coefficient at impact is given with:

9 $$\begin{aligned} \beta _1=\frac{\rho _0}{\rho _1}\left( \kappa _1+\frac{{\rm Fr}^2}{2}\right) \end{aligned}$$

For a debris flow treated as a homogeneous fluid, i.e. with $\kappa _0=\kappa _1=1$ and $\rho _1=\rho _0$, Eq. (7) becomes:

10 $$\begin{aligned} \beta _2=1+\frac{{\rm Fr}^2}{2} \end{aligned}$$

Equation (10) is commonly referred to as the frictionless point-mass (PM) model [25, e.g.] and often used to predict the run-up height of debris flows against vertical walls [8, 30].

Due to the abrupt change in momentum when a debris flow hits a vertical barrier, the non-dimensional run-up coefficient $\beta$ can also be estimated by considering mass and momentum balance of a control volume. The concept is based on a reflecting wave approach at impact [14, 26, 52], and the corresponding theoretical model is known as the momentum jump (MJ) model [25]. Also, the MJ model accounts in its most elaborate form for the possibility of normal stress anisotropy ($\kappa$) and changes in bulk densities of the incoming flow ($\rho _0$) and at impact ($\rho _1$):

11 $$\begin{aligned} \frac{\rho _1}{\rho _0}\beta ^2-\beta -1+\frac{\rho _0}{\rho _1}\beta ^{-1}-\frac{2}{\kappa }{\rm Fr}^2=0 \end{aligned}$$

It has to be noticed that the coefficient $\kappa$—included in the MJ model in its most explicit form (Eq. 11)—does not differ between incoming flow and impact.

By assuming $\kappa\,\approxeq\,\kappa _1$ and converting Eq. (11) into a cubic form, an analytical solution can be given based on Cardano’s formulae:

12 $$\begin{aligned} \beta _3=2\root 3 \of {r}\cos \zeta +\frac{\rho _0}{3\rho _1} \end{aligned}$$

12a $$\begin{aligned} r&=\sqrt{-\left( \frac{e}{3}\right) ^3} \end{aligned}$$

12b $$\begin{aligned} e&=\frac{-3\left( \frac{\rho _1}{\rho _0}\right) \left( 1+\frac{2{\rm Fr}^2}{\kappa _1}\right) -1}{3\left( \frac{\rho _1}{\rho _0}\right) ^2} \end{aligned}$$

12c $$\begin{aligned} \zeta&=\frac{1}{3}\arccos \left( \frac{-q}{2r}\right) \end{aligned}$$

12d $$\begin{aligned} q&=\frac{27\left( \frac{\rho _1}{\rho _0}\right) -9\left( \frac{\rho _1}{\rho _0}\right) \left( 1+\frac{2{\rm Fr}^2}{\kappa _1}\right) -2}{27\left( \frac{\rho _1}{\rho _0}\right) ^3} \end{aligned}$$

Equation (11) and its analytical solution (Eq. 12) is equivalent to equation A7 in [25] and equation 6 in [33]. Both studies provide a detailed derivation.

For a debris flow treated as a homogeneous fluid, i.e. with $\kappa =1$ and $\rho _1=\rho _0$, Eq. (11) becomes equivalent to equation 2 in [4] and is the base of the impact equation 8 in [53]. Applying $\kappa =1$ and $\rho _1=\rho _0$, the MJ model (Eq. 11) yields to:

13 $$\begin{aligned} \beta _4'=[1+(2{\rm Fr}^2 )^n ]^\frac{1}{2n} \end{aligned}$$

with $n=1/2$ proposed by [53]:

13a $$\begin{aligned} \beta _4''=1+\sqrt{2}{\rm Fr} \end{aligned}$$

respectively $n=3/5$ proposed by [3]:

13b $$\begin{aligned} \beta _4=(1+1.51{\rm Fr}^{1.2})^{5/6} \end{aligned}$$

The difference between the two approaches is marginal and we rely in this study on Eq. (13b) for the prediction of $\beta _4$.

### Scientific challenges and research objectives

We assume that the simple impact model (Eq. 6) allows the prediction of pressure peaks of slow granular debris flows, whose flow condition is largely dominated by frictional or collisional flow resistance. For its application, the incoming flow height $h_0$, the stress anisotropy $\kappa _1$, the bulk flow density $\rho _1$ and the maximum run-up (i.e. run-up coefficient $\beta$) at impact must be known. While the concepts to estimate the dimensional run-up coefficient $\beta$, either by energy or mass and momentum conservation, have their physical justifications, work on expected stress anisotropy $\kappa _1$ and bulk density $\rho _1$ ratios at impact is rather rare.

For this reason, we conducted small-scale debris-flow experiments, focussing on the determination of peak impact pressures ($p_{{\rm peak}}$), stress anisotropy ($\kappa _1$) and density variations ($\rho _0,\rho _1$) at impact for two debris-flow mixtures. Both mixtures were chosen to ensure that the contrasting prototypical debris flows show either a friction-induced or collision-induced flow resistance type. To better account for uncertainties, 17 replicates per mixture were carried out. We further provide data on measured inflow bulk densities ($\rho _0$) from three runs per mixture not being influenced by the impact measurement device. For all replicates, we also determined normal forces ($F_N$) and pore water pressures ($p_f$) at exactly the same location, measured with a newly developed device just before impact and without impact, respectively. Finally, back calculated run-up coefficients from measured peak impact pressures are compared with different theoretical run-up models (Eqs. 9, 10, 12 and 13b).

## Methodology

### Experimental set-up

**Fig. 3:**
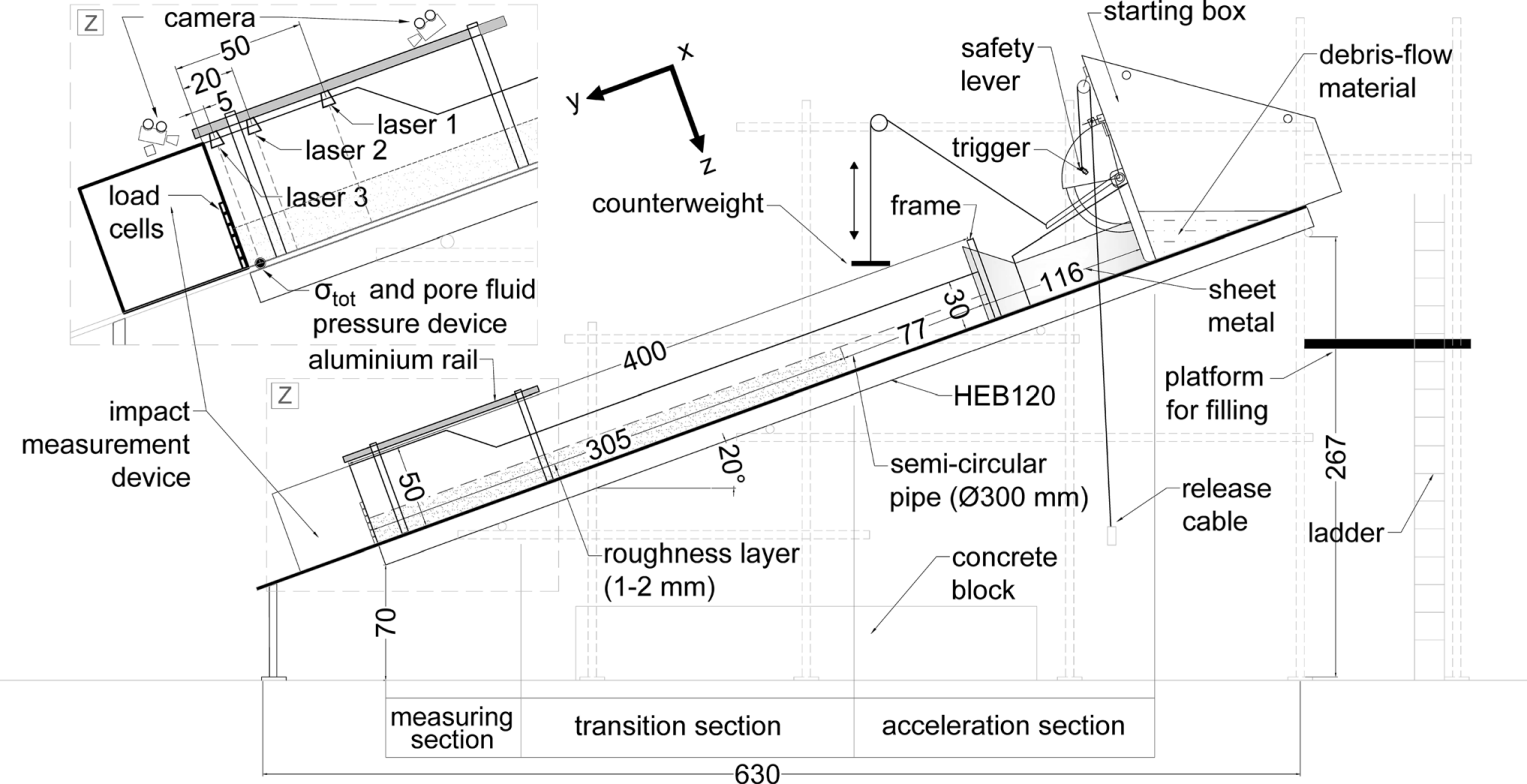
Experimental setup and detailed sketch of the measuring section, length dimensions in cm

The experimental set-up (Fig. 3) consists of a 400-cm long semi-circular channel with a diameter of 30 cm and an inclination of 20 °C that is framed by wooden formwork panels and that is mounted on two HEB120 steel beams with a length of 600 cm each. The starting box, a rectangular reservoir, contains the debris-flow material and is accessible via a ladder and platform for filling. By pulling the release cable, the safety lever is released, and the rubber-band cushioned counterweight abruptly pulls the flap of the start box open, releasing the debris-flow material into the channel. Simultaneously, the trigger starts the measurements. The debris-flow material first enters a 193-cm long section for acceleration that is composed of 116 cm sheet metal and 77 cm of the bare semi-circular drainage pipe. This section is followed by a 305-cm transfer and measuring section which is covered with a roughness-layer of grain diameters ranging from 1 mm to 2 mm. All measurements are taken within the last third of the flume (measuring section, Fig. 3).

Slope, length and roughness of the channel in combination with defined sediment mixtures were chosen to ensure steady flow conditions at the end of the flume, i.e. in the measuring section. This is necessary, because the considered analytical impact model assumes a steady, uniform incident flow. The experimental setup was also designed in such a way that the analysis of the measurements is not disturbed by the self-resonance of the entire experimental construction.

### Experimental design and debris-flow mixtures

**Fig. 4:**
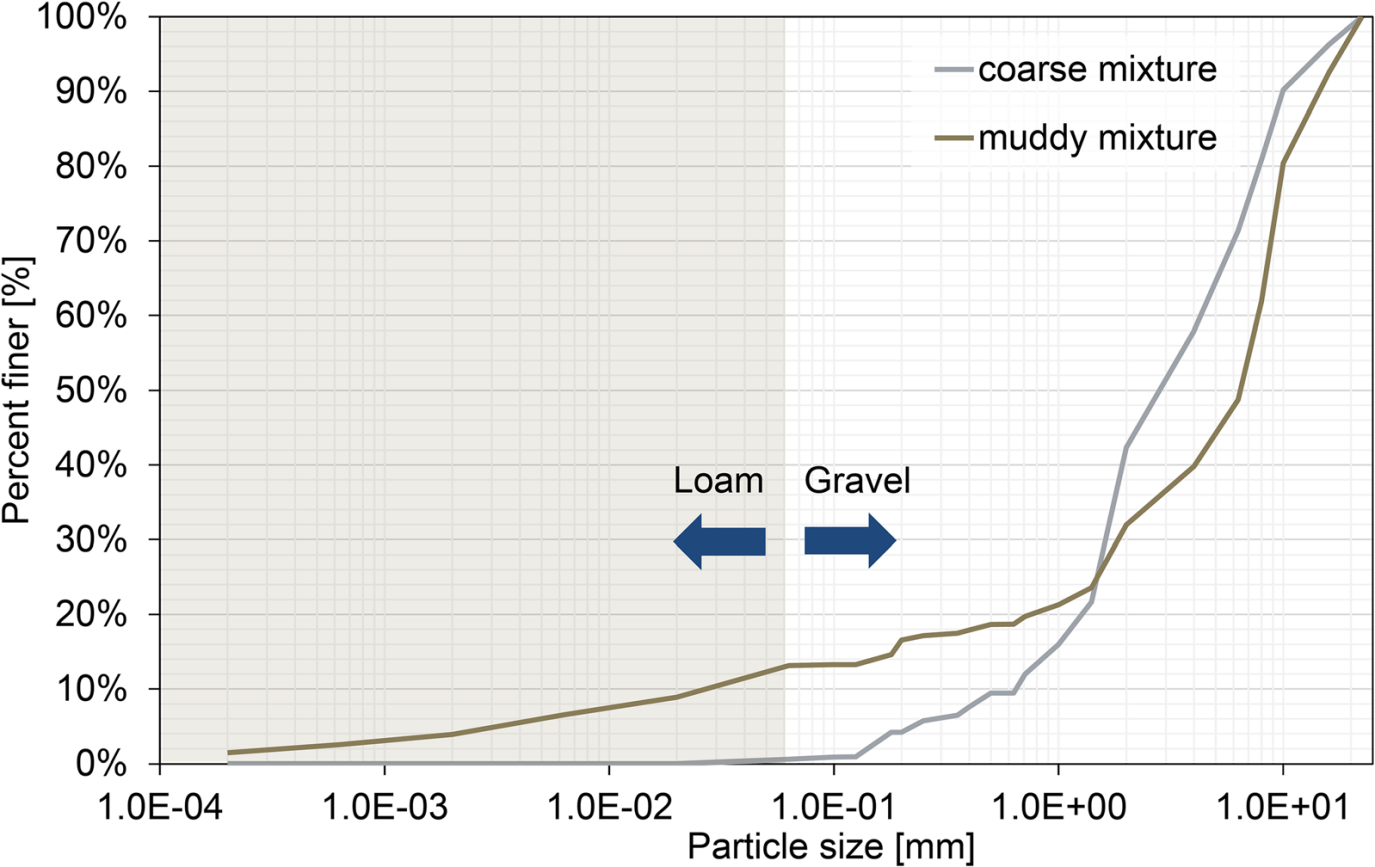
Grainsize distributions of the coarse and muddy mixture

In response to the wide range of natural flow properties of debris flows, we conducted experiments with two different debris-flow mixtures, either “coarse” or “muddy” in nature (Fig. 4).

Both mixtures are based on the experience of previously conducted small-scale experiments of debris flows with angular natural materials [44, 45].

The coarse mixture consists exclusively of non-cohesive material and lacks any silt and clay. By an increase of gravel concentration—while maintaining the same water concentration? An increase in grain frictional effects for the coarse mixture is expected.

The muddy mixture differs from the coarse mixture by a high fraction of fine particles and a low coarse grain fraction. By increasing the fine particle content in the pore water of the muddy mixture, we expect an increase in viscous stress. The share of the total fine particles for the muddy mixture consists of natural loam with an average of 14.7% sand, 57.7% silt and 27.4% clay, agreeing quite well with the finest particle content of debris-flow compositions in the study of [11]—leading to the longest runouts and highest flow velocities.

For both mixtures, the released total mass of 50  kg was kept constant for all experiments. To keep a constant bulk density at rest ($\rho _b=1940$ kg m^-3^), the coarse mixture consisted of 68% sediment and 32% water by volume, while the muddy mixture had a slightly higher sediment to water ratio of 69% sediment to 31% water by volume.

In this study, ten replicates ($\#$:1–10) of the coarse mixture and seven replicates ($\#$:11–17) of the muddy mixture were conducted to measure impact-induced horizontal forces, flow heights, normal stresses, as well as bulk densities. For each of those replicates, the observed stress anisotropy coefficient ($\kappa _1$) and flow density ($\rho _1$) at impact were derived at the time of the maximum impact pressure. To distinguish from values predicted by the impact model (Eqs. 4 and 6), we have denoted the maximum impact pressures derived from measurements with $p_{\max }$.

In addition, three replicates based on the coarse mixture ($\#$:18–20) and three replicates based on the muddy mixture ($\#$:21–23) were conducted without mounting the impact measurement device to the flume, to determine unaffected flow heights, normal stresses as well as bulk densities before impact. All measured as well as back-calculated values are given within the Tables 2 and 3, provided in the Appendix. An overview of mean values and standard deviations of the most important results are listed in Table 1.

### Scaling considerations

Complete dynamic similarity of all forces acting in nature and in a physical debris-flow model is not feasible by using the same fluid with the same viscosity [5, 12, 21, 45, 48, e.g.]. However, several studies indicate that there are considerable differences regarding the origin of the bulk resistance of natural debris-flow events [10, 27, 54]. In contrast to previous approaches, where observed events act as a basis for the experiments [10, 12, 49, 51], our debris-flow prototypes are not a back-modelling, but an attempt to represent two prototypical debris-flow processes.

**Fig. 5:**
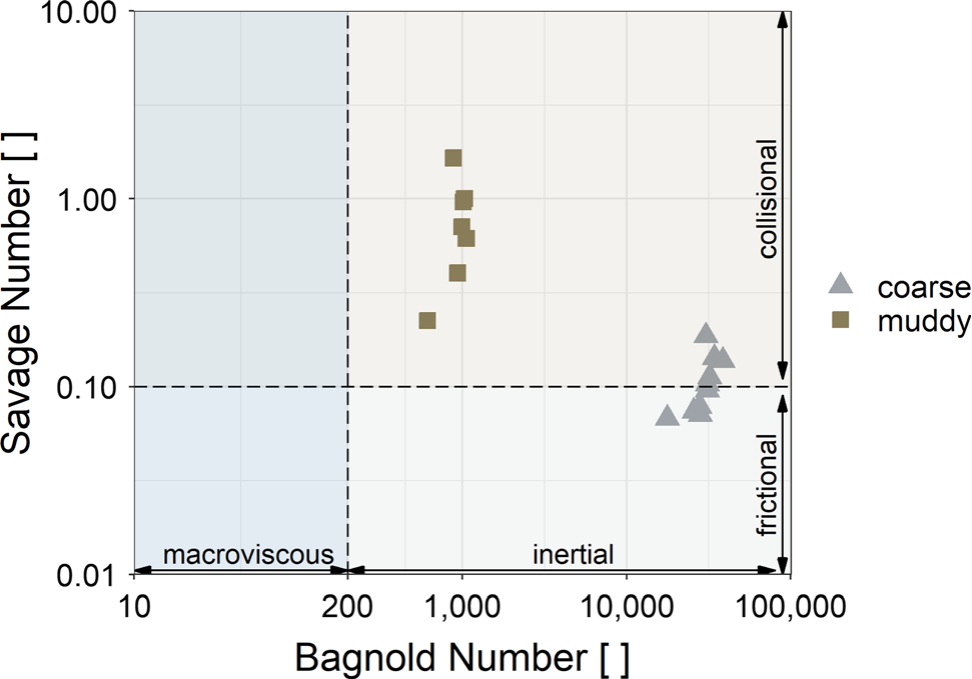
Savage number against Bagnold number to identify prototypical flow properties given for all replicates, either based on coarse or muddy debris-flow mixtures

To identify prototypical flow properties of experimental debris flows, dimensionless numbers such as the Savage or Bagnold number can be used (Fig. 5). The Savage number $N_S$ relates the inertial shear stress caused by grain collision with the inertial shear stress caused by friction from permanent grain contact.

14 $$\begin{aligned} N_S=\frac{\rho _s {\dot{\gamma }}^2 d}{\sigma _{{\rm eff}}} \end{aligned}$$

Equation (14) includes the density of the solid particles ($\rho _s$), the shear rate (${\dot{\gamma }}$), a characteristic grain diameter (*d*) and the effective normal stress ($\sigma _{{\rm eff}}$). Savage and Hutter [42] propose a threshold value of $N_S\approx 0.1$, to differ friction-dominated flows from collisional-dominated flows. The Bagnold number $N_B$ describes the ratio of the flow resistance due to grain collision with the flow resistance due to the viscosity of the liquid portion of the debris-flow mixture.

15 $$\begin{aligned} N_B=\frac{C_V}{1-C_V}\frac{\rho _s {\dot{\gamma }} d^2}{\eta _f} \end{aligned}$$

In addition to $\rho _s$, ${\dot{\gamma }}$ and *d*, Eq. (15) takes into account solid concentration of the debris mixture ($C_V$) as well as fluid viscosity ($\eta _f$).

To estimate $N_S$ as well as $N_B$ of the conducted experiments, we assume the shear rate as a linear change of velocity over the flow height (${\dot{\gamma }}=v_0/h$). While the fluid viscosity for the coarse mixture corresponds to the fluid viscosity of water ($\eta _f={0.001\,{\text{Pa s}}}$), we estimated it for the muddy mixture with a Bohlin Visco 88 viscometer ($\eta _f={0.09\,{\text {Pa s}}}$).

According to the range of $N_B$, the flow behaviour seems to be governed by the grain interaction in all our experiments. However, the bulk resistance caused by the grain interaction is rather influenced by friction in coarse mixtures ($N_S<0.1$), whereas the collisional grain interaction dominates in muddy mixtures ($N_S<0.1$). Similar as discussed in Sanvitale and Bowman [41], the relatively high Savage numbers are based on the characteristic grain size, which in this study was given with $d_{50}={5\,{\text {mm}}}$ for coarse and $d_{50}={3\,{\text {mm}}}$ for muddy mixtures. Thus, Fig. 5 primarily reflects the range of gravel fraction interaction for the muddy mixture.

### Measuring devices and signal analyses

**Fig. 6:**
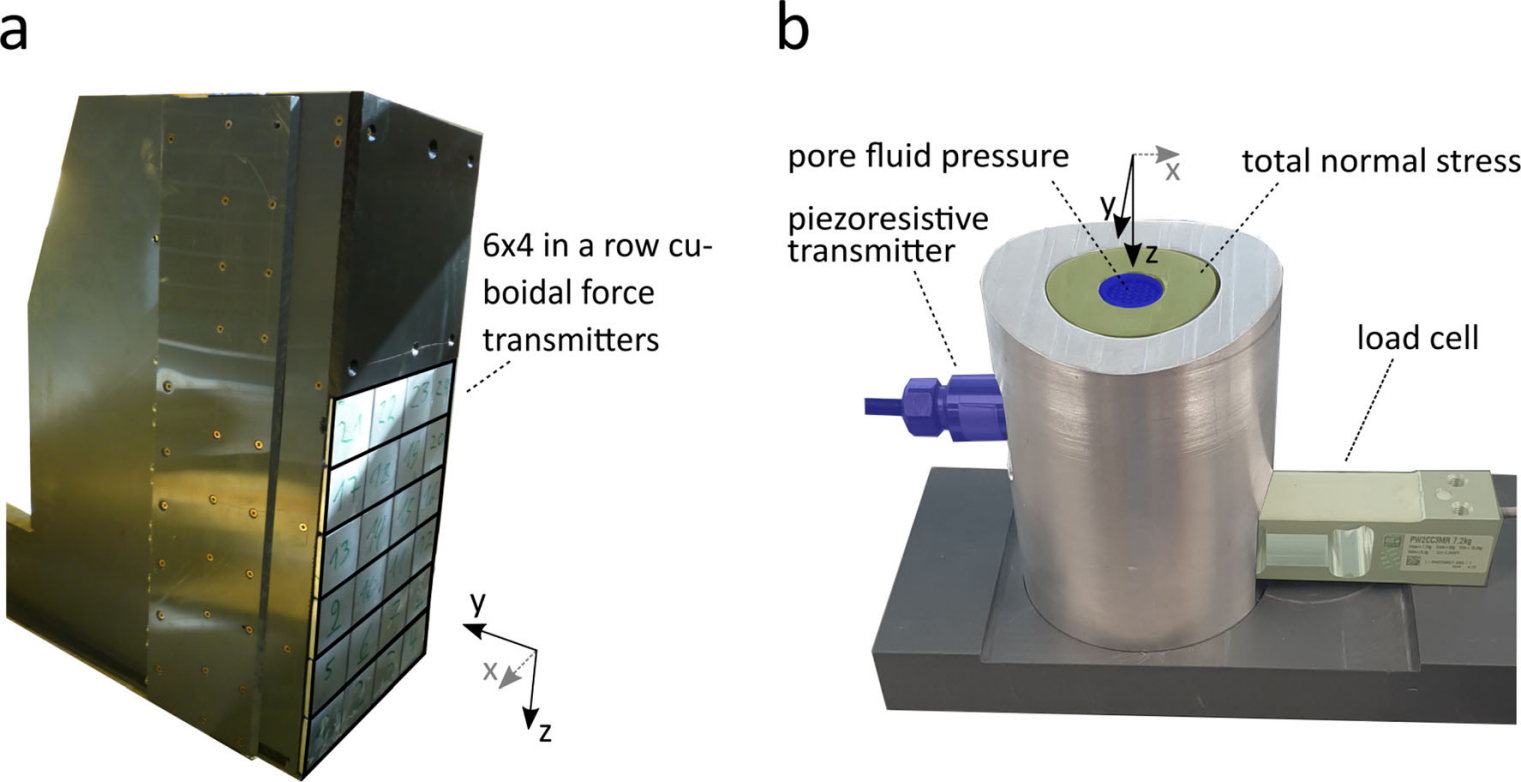
Devices used for measuring: **a** horizontal impact forces and **b** total normal force and pore fluid pressure, respectively

Direct measurements in this study included flow height, pore fluid pressure, total normal stress, and horizontal impact forces. The measurement signal was acquired with a Quantum MX1601B datalogger from HBM and postprocessed via the corresponding software ’catman V5.3.2’. A measurement frequency of 2.4 kHz was chosen. For the determination of maximum values of horizontal impact pressure, total normal stress and pore fluid pressure, the logged signals were post-filtered by applying a Butterworth lowpass ?lter with a cutoff frequency of ${10\,{\text {Hz}}}$. Random effects such as resonance frequency of the flume or the impact measurement device could be identified and excluded in frequency ranges above the cutoff frequency. Three laser devices were installed at different distances along the channel to measure flow heights (c.f. Figure 3). The maximum front velocity ($v_0$) was estimated based on the time when the debris-flow front of the replicate passed the laser devices 1 and 2—similar to the approach described in [44]. The maximum flow heights $h_0$ were based on the measurements from laser device 1, as these are unaffected by the impact. A characteristic Froude number of the approaching flow was then calculated, based on Eq. (7). Additionally, two highspeed cameras (120 frames per second) were mounted on the flume, one facing the starting box and one facing the impact measurement device. The cameras were used exclusively for a visual documentation of the process. Basal total normal stresses ($\sigma _{{\rm tot}}$) were derived from the normal forces ($F_N$), measured with a load cell attached to a construction specifically designed for this study. The new device allows to measure basal pore fluid pressures ($p_{f}$) at exactly the same measuring location of $\sigma _{{\rm tot}}$, by integrating a piezoresistive pressure transmitter within the load cell (Fig. 6b). The piezoresistive pressure transmitter was mounted with a 2-mm mesh to avoid clogging of the sensor and to measure the rapid pore water pressure changes during flow and impact. Similar measurement systems have been used in the field to measure pore fluid pressure [35]. After each run, the sensors were cleaned and filled with clean water for the next replicate to provide an accurate measurement. Laser device 3, which was mounted exactly above the new device, measured the corresponding flow heights to the measured normal forces as well as the pore water pressures.

Horizontal impact forces were measured with the same impact measurement device as used in [44]. The impact measurement device (Fig. 6a) consists of 24 cuboidal aluminium load cells ($6 \times 4$ in a row), each with an area of $0.04\,{\rm m}\times 0.04\,{\rm m}$, and was placed directly at the end of the flume to provoke a complete impact.

## Results

### Maximum impact pressures

We derived the maximum impact pressure for each replicate ($p_{\max }$), by summarising measured forces in adjacent load cells and dividing them by the corresponding area of the impact measurement device (Fig. 5a). The progression of the pressure values per row over time (Figs. 15, 16) showed that $p_{\max }$ was constantly measured within the lower load cells of the impact measurement device, for all replicates.

**Fig. 7:**
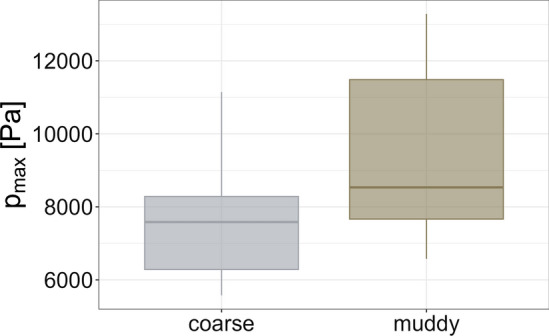
Distributions of measured maximum impact pressures ($p_{\max }$) for replicates based on the coarse or muddy mixture, respectively

The difference in flow dynamics related to the coarse and muddy mixture is also reflected by the maximum impact pressure values obtained for all replicates. As shown in Fig. 7, the resulting $p_{\max }$ values differ between the coarse and muddy mixture, with higher values related to muddy based replicates. Mean values and standard deviations of $p_{\max }$ values are listed in Table 1.

**Table 1:** Mean values and standard deviations of measured maximum impact pressures ($p_{\max }$), liquefaction ratios (${\rm LR}$), anisotropic stress coefficients ($\kappa _1$), bulk densities at impact ($\rho _1$) as well as bulk density ratios ($\rho _0$) for each mixture

Mixture	$p_{\max }$ (Pa)	LR (–)	$\kappa _1$ (–)	$\rho _1$ (kg m^-3^)	$\rho _0$ (kg m^-3^)
Coarse	7558	0.60	2.66	2114	1791
	$\pm 1560$	$\pm 0.06$	$\pm 0.90$	$\pm 694$	$\pm 20$
Muddy	9528	0.73	1.53	2204	1720
	$\pm 2352$	$\pm 0.08$	$\pm 0.26$	$\pm 464$	$\pm 122$

### Bulk flow, basal-, and non-hydrostatic longitudinal normal stresses

Observations of real debris-flow events suggest that due to the frictional properties, granular debris-flow mixtures have a high internal strength and thus their deformation behaviour and subsequently their force transfer differs significantly from debris flows with dominantly viscous properties. Such a difference in bulk resistance can be described with the liquefaction ratio as the ratio of pore fluid pressure ($p_{f}$) to total normal stress ($\sigma _{{\rm tot}}$):

16 $$\begin{aligned} {\rm LR}=\frac{p_{f}}{\sigma _{{\rm tot}}} \end{aligned}$$

The range of the liquefaction ratio is theoretically between zero and one. When pore pressure equals normal stress (${\rm LR}=1$), the mass is fully liquefied. This condition refers to a fluid-like behaviour of the mass and is considered as the theoretical upper limit of the liquefaction ratio. When ${\rm LR}=0$, particles in the mixture are not supported by any pore fluid and external load is fully transmitted to the grain contacts. This condition refers to a dry granular flow.

For saturated mixtures, where the timescale of pore pressure diffusion is shorter than the timescale of deformation, fluid pressure equals the hydrostatic pressure [23]. In that case, *LR* typically ranges between 0.4 and 0.6 [28]. For grain-fluid mixtures having a wide grain size distribution, fluid pressure in excess of hydrostatic conditions is expected to occur.

**Fig. 8:**
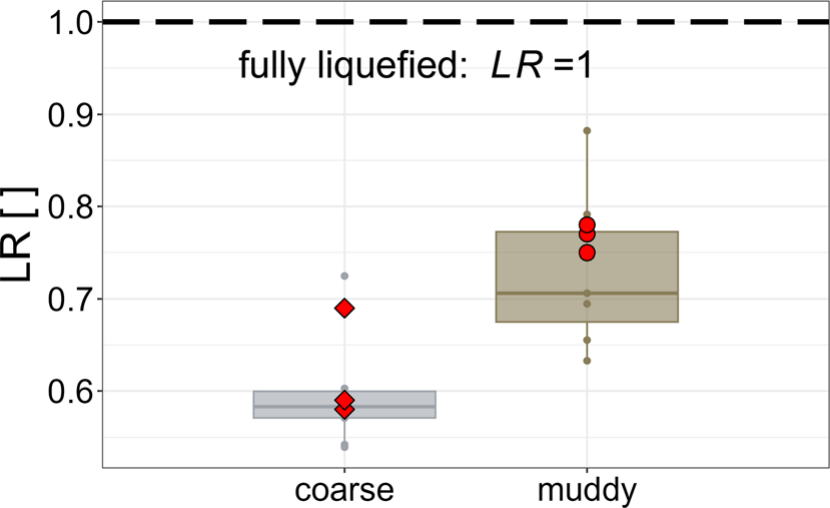
Distributions of liquefaction ratios (${\rm LR}$) for replicates based on the coarse or muddy mixture, respectively. The red points in refer to liquefaction ratios of the unaffected replicates $\#$:18–20 and $\#$:21–23, i.e. experimental runs with no impact measurement device mounted at the end of the flume. The dashed line refers to the theoretical limit of the liquefaction ratio, defined with ${\rm LR}=1$

Our results show that estimated liquefaction ratios differ between the experimental material mixtures (Fig. 8).

In general, lower liquefaction ratios are achieved for replicates of the coarse mixture—in contrast to the replicates based on muddy mixture, which also show a higher variability (c.f. Table 1). Compared to the coarse mixture, replicates of the muddy mixture generally had higher velocities. We attribute this to the presence of silt and clay, which leads to an increase of pore fluid pressure and associated reduction of frictional flow resistance of the coarse sediment [24].

The determination of the characteristic stress anisotropy or (earth) pressure coefficient ($\kappa _1$) for each impact related replicate is calculated with Eq. (17) at the time of measured peak impact pressure ($p_{\max }$):

17 $$\begin{aligned} \kappa _1=\frac{\sigma _{{\rm tot}}}{p_{\max }} \end{aligned}$$

**Fig. 9:**
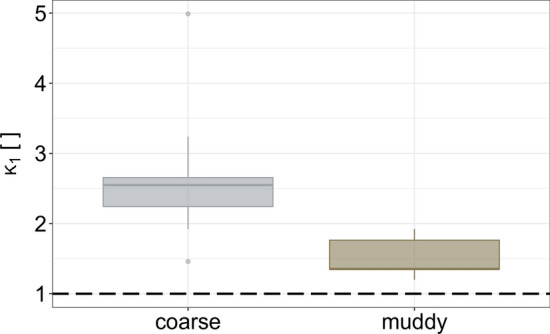
Distributions of anisotropy coefficients ($\kappa _1$) for replicates based on the coarse or muddy mixture, respectively. The dashed line indicates hydrostatic pressure conditions with $\kappa _1=1$

In Fig. 9, distributions of compiled anisotropy coefficients $\kappa _1$ of all impact related replicates per mixture are shown.

Our experiments reveal significantly higher anisotropic behaviour—related to hydrostatic pressure conditions—for the replicates based on the coarse mixture. While replicates corresponding to the muddy mixture are relatively close to a hydrostatic pressure distribution at impact—indicated with $\kappa =1$ in Fig. 9. Mean values and standard deviation of $\kappa _1$-values are listed in Table 1.

### Bulk densities

The estimation of bulk densities are based on Eq. (18), relating the measured normal force ($F_N$) and the corresponding flow height (*h*) of the experimental debris flows to $A_S=0.002\,{{{\rm m}}^2}$, the sensor area of the normal force measurement device (Fig. 5), and *g*, the acceleration due to gravity.

18 $$\begin{aligned} \rho =\frac{F_N \cos \theta }{ghA_S} . \end{aligned}$$

The relevant flow heights are all based on laser measurements (Laser 3).

**Fig. 10:**
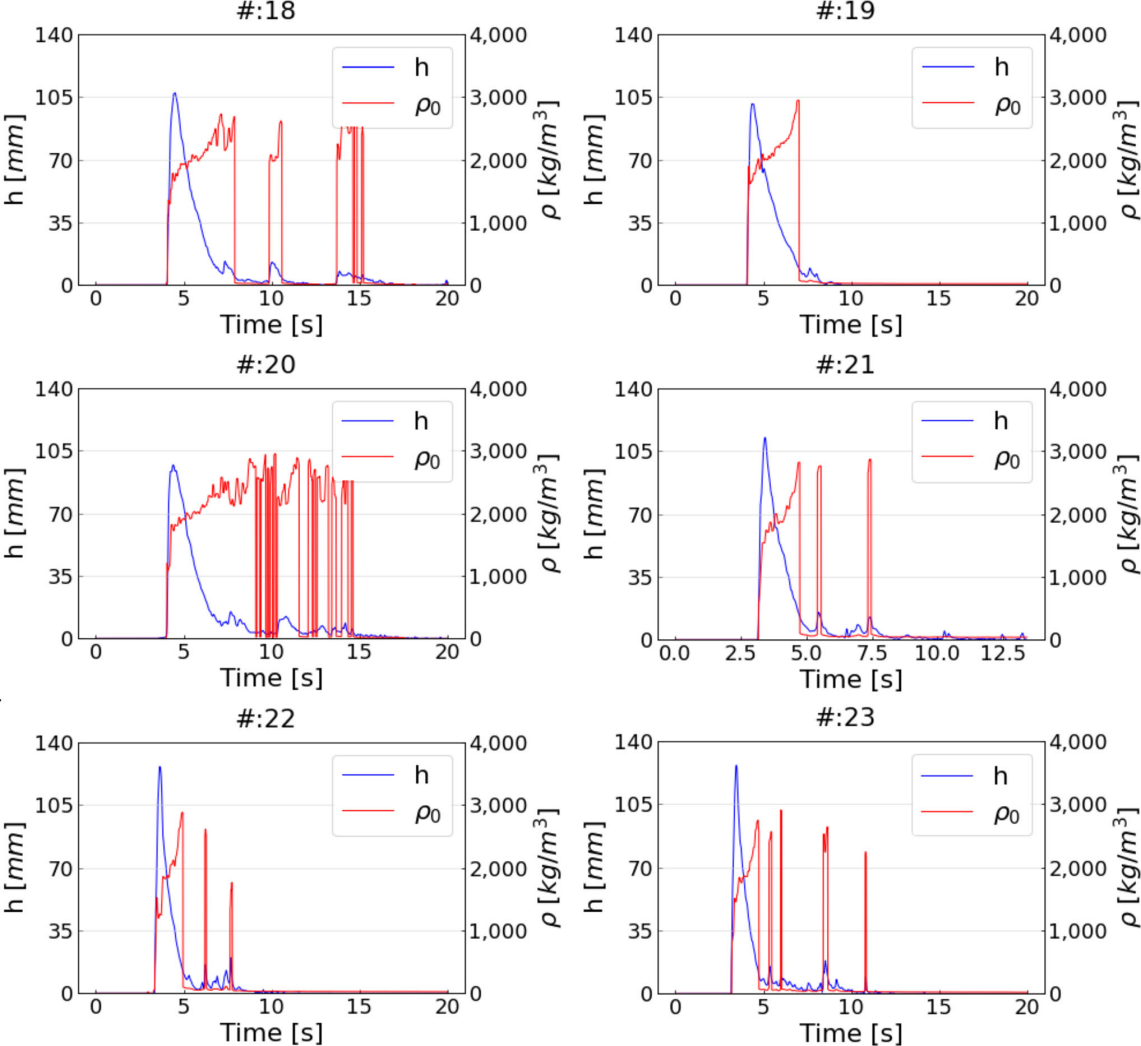
Inflow bulk densities ($\rho _0$) as well as corresponding flow heights (*h*) over time for the unaffected coarse as well as unaffected muddy replicates

Incoming flow densities before impact ($\rho _0$) are shown in Fig. 10 and are based on $F_N$ as well as *h* values of the unaffected replicates. Here, the applied *h* values correspond to the incoming flow heights $h_0$.

Regardless of their mixtures, the experimental debris flows not affected by impact showed lower density values at the debris-flow front and higher density values in the liquefied tail of the flow. However, for this study, we averaged the inflow bulk densities ($\rho _0$) at the time of the maximum flow height. Mean values of $\rho _0$ for all replicates are listed in Table 1.

The relevant flow height to determine the flow density at impact ($\rho _1$) corresponds to $F_N$ and *h* values at the time of the maximum impact pressure ($p_{\max }$). The applied flow height *h* is, however, not necessarily equal to $h_1$, as the basal normal force is measured 5 cm away from the impact measurement device for technical reasons. Mean values of $\rho _1$ for all replicates are listed in Table 1.

### Run-up coefficient

Transforming Eq. (6), the run-up coefficient $\beta$ can be back-calculated by applying the corresponding maximum impact pressure ($p_{\max }$), the stress anisotropy coefficient $\kappa _1$ as well as the density $\rho _1$ for each impact related replicate:

19 $$\begin{aligned} \beta ^*=\frac{p_{\max }}{\kappa _1\rho _1 g h_0 \cos \theta }. \end{aligned}$$

**Fig. 11:**
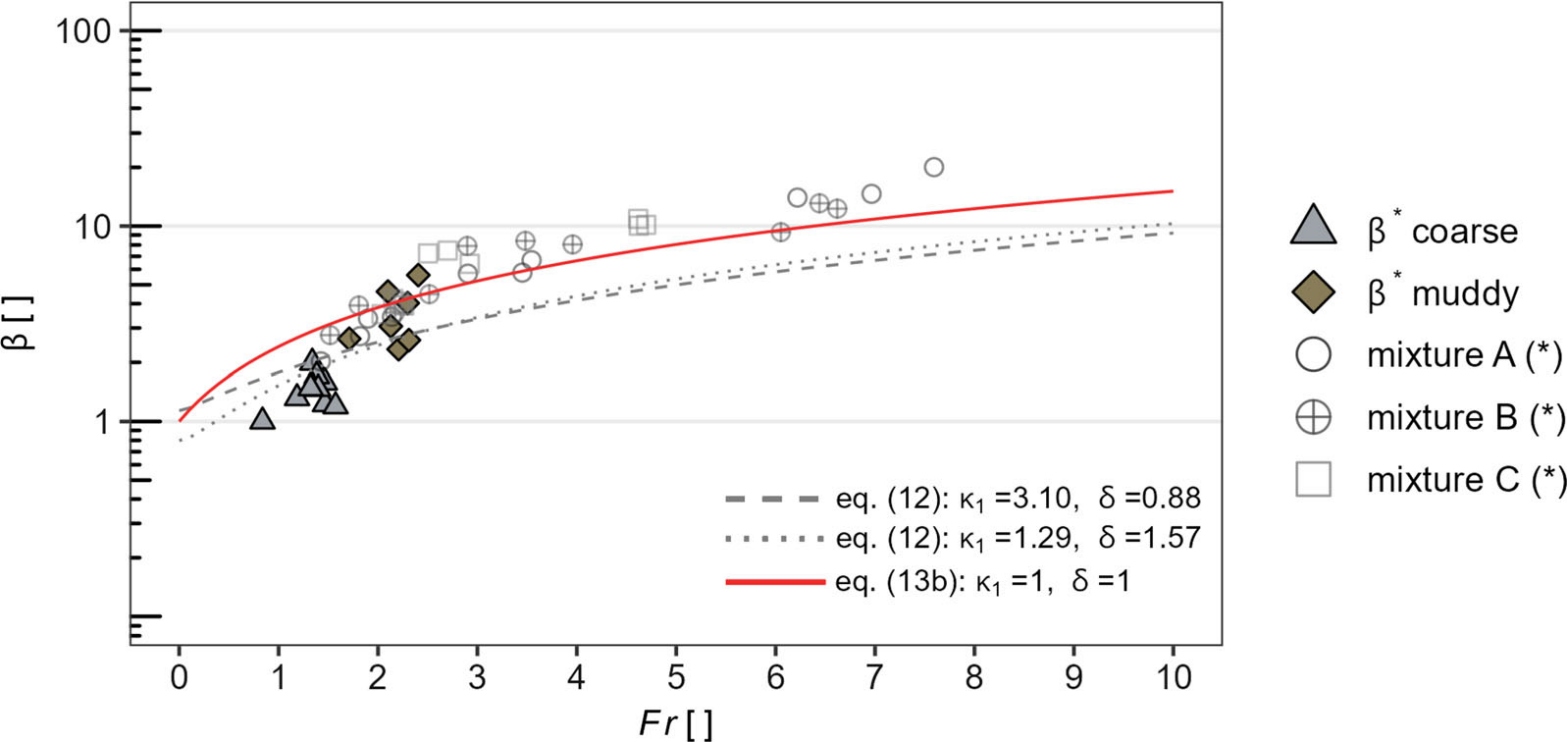
Back-calculated run-up coefficients $\beta ^*$, based on Eq. (18), as a function of the Froude number (${\rm Fr}$), together with results of run-up experiments against a vertical wall from [40]—denoted with (*). The solid line correspond to the frictionless-point mass model (Eq. 10). The dashed or dotted lines refer to Eq. (9), taking into account one standard deviation from the means of the measured $\kappa _1$ as well as bulk flow density variations, given with $\delta =\rho _1/\rho _0$

**Fig. 12:**
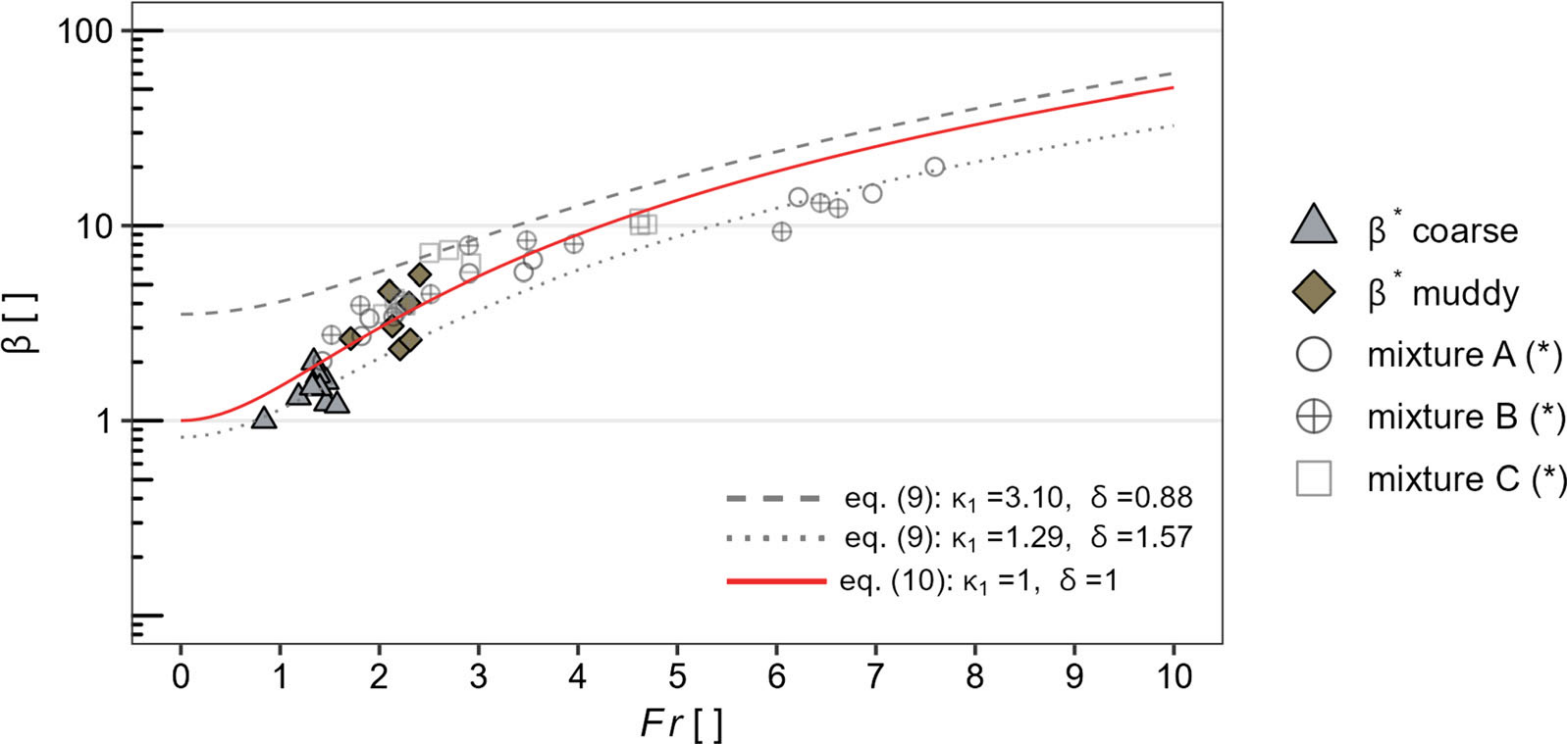
Back-calculated run-up coefficients $\beta ^*$, based on Eq. (18), as a function of the Froude number (${\rm Fr}$), together with results of run-up experiments against a vertical wall from [40]—denoted with (*). The solid line correspond to the momentum jump model for homogeneous fluids (Eq. 13b). The dashed or dotted lines refer to Eq. (12), taking into account one standard deviation from the means of the measured $\kappa _1$ as well as bulk flow density variations, given with $\delta =\rho _1/\rho _0$

Figures 11 and 12 relate back-calculated run-up coefficients ($\beta ^*$) based on Eq. (19) to the Froude number (${\rm Fr}$), together with measured run-up coefficients of experiments against a vertical wall from [40].

In Fig. 11, the results are further compared with run-up coefficients, predicted on the basis of energy conservation. Here, the solid red line correspond to the frictionless-point mass model (PM), according to Eq. (10). The grey dashed or dotted lines show the possible range of predicted $\beta$ values based on Eq. (9), taking into account one standard deviation from the means of the measured stress anisotropy values ($\kappa _1$) as well as bulk flow density variations, given with $\delta =\rho _1/\rho _0$.

In Fig. 12, $\beta$ values, predicted by models considering mass and momentum conservation at impact, are shown. Here, the solid red line correspond to the momentum jump model for homogeneous fluids (Eq. 13b). The dashed and dotted grey lines correspond to the momentum jump model in its most elaborate form (Eq. 12), accounting for one standard deviation from the means of the measured stress anisotropy values ($\kappa _1$) as well as bulk flow density variations, given with $\delta =\rho _1/\rho _0$.

Although the back-calculated $\beta ^*$ values are basically only valid for granular, i.e. frictional or collisional induced debris flows, they approximate very well to the experiments of [40]. Their mixtures A, B and C consist of 60% sediments per volume with decreasing clay content, i.e. mixture C is the most granular and mixture A the most muddy mixture.

The prediction models based on the energy conservation approach (Fig. 11) appear to cover the observed run-up coefficients over a wide Froude range, compared to the prediction models based on the momentum jump approach (Fig. 12). Taking into account stress anisotropy and variations in flow density, $\beta$ values estimated on the basis of Eq. (12) show a high variance especially in low Froude areas (Fig. 11). In particular, the influence of stress anisotropy seems to be crucial in this case. However, it is important to emphasise that due to the unknown stress anisotropy of the incoming bulk flow, and thus with the assumption $\kappa _0=1$, the estimated run-up coefficients represent maximum values.

## Discussion

For granular debris flows which show low energy dissipation due to smooth gradual run-up, knowledge of anisotropic normal stress and flow density at impact has to be considered to predict peak impact pressures.

In our study, measured anisotropic stress coefficients for prototypical debris flows based on the muddy mixture, range between $\kappa _1=1.35$ and $\kappa _1=1.92$. For prototypical debris flows based on the coarse mixture the measured anisotropic stress coefficients show higher variability and range between $\kappa _1=1.46$ and $\kappa _1=4.99$. Those values seem plausible as they (i) are greater than unity—caused by compression on impact [18, 36], and (ii) reflect the differences of the applied mixtures through the different earth pressure state.

Measured bulk densities of the incoming ($\rho _0$) and impacted ($\rho _1$) flow differ significantly from the bulk density at rest ($\rho _b = 1.940$ kg m^-3^). Here, prototypical debris flows based on the coarse mixture have lower density ratios ($\delta =\rho _1/\rho _0=1.18$) when compared to the density ratios of the muddy based replicates ($\delta =1.28$), suggesting that these prototypical debris flows are denser by nature. However, our results also indicate that incoming bulk flow densities increase from the matrix-supported, granular head to the more fluid-related tail of the debris flow.

Another decisive factor for the determination of pressure peaks caused by the impact of debris-flow processes on obstacles is the dimensionless run-up coefficient $\beta$. Either assuming conservation of energy or mass and momentum balance, it is possible to calculate potential run-up heights in relation to the dynamic of flow properties.

Based on energy conservation, the frictionless point mass (PM) model predicting run-up coefficients by assuming $\kappa _1=1$ and $\delta =1$ (Eq. 10) appears to fit quite well, both the back-calculated run-up coefficients based on the muddy related replicates of this study as well as the results of [40]. In the lower Froude range, however, the model tends to overestimate the back-calculated run-up coefficients for the coarse mixture based replicates. A closer fit is likely, if stress anisotropy as well as bulk density variations are considered (Eq. 9).

**Fig. 13:**
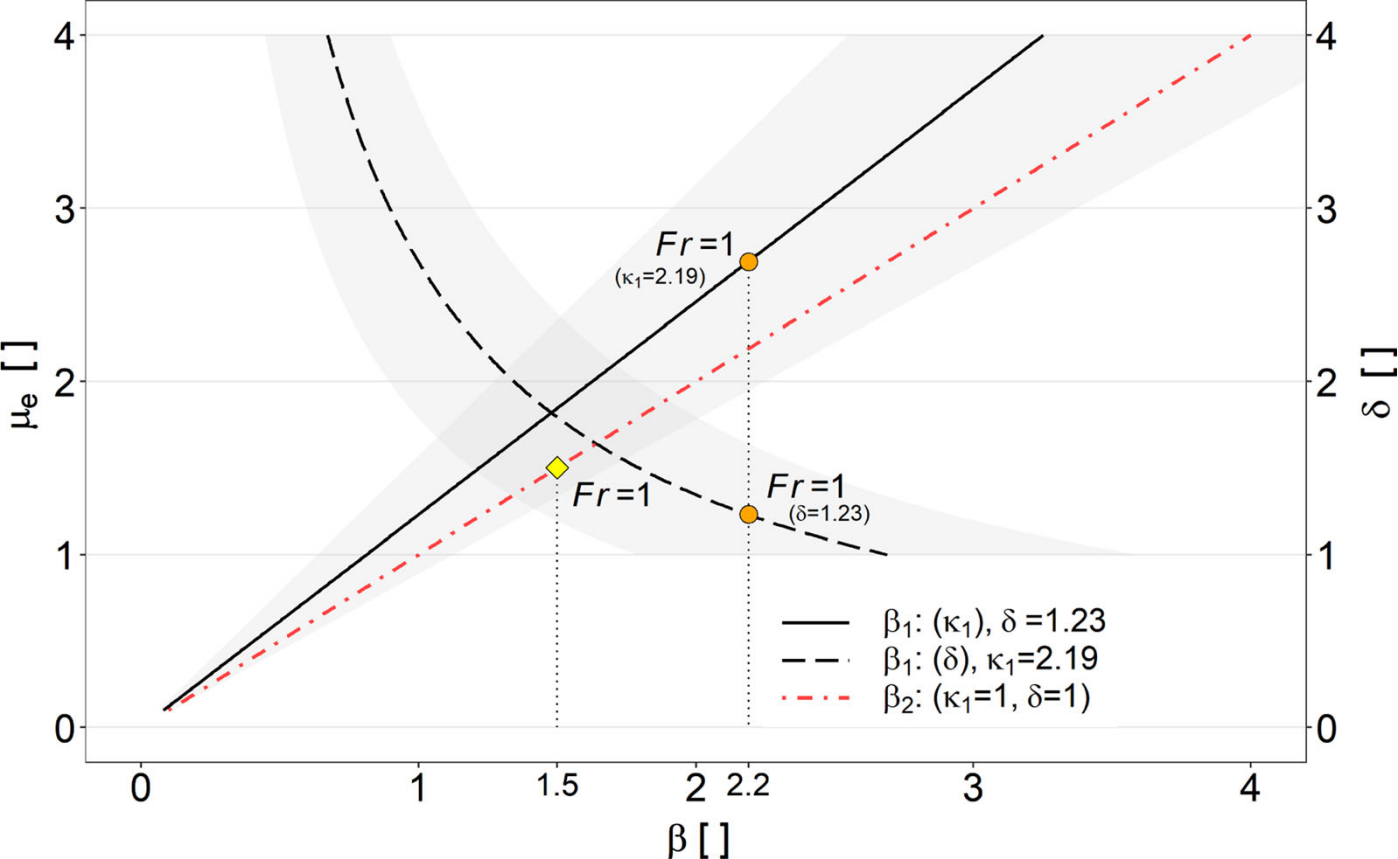
Partial dependency plot of $\mu _e$ (respectively $\kappa _1$) and $\delta$ for estimating the run-up coefficient $\beta _1$ when accounting for energy conservation (Eq. 9). The fixed values correspond to the respective mean values, $\langle \kappa _1\rangle =2.19$ or $\langle \delta \rangle =1.23$ of all conducted impact related replicates. The range of uncertainty is based on one standard deviation from the fixed mean values. Also shown is the influence of $\mu _e$ respectively $\kappa _1=1$ on the calculation of the run-up coefficient $\beta _2$, when based on Eq. (10). The markers correspond to the run-up coefficients valid at ${\rm Fr}=1$

The influence of stress anisotropy ($\kappa _1$) as well as density variation ($\delta$) on the estimation of the run-up coefficient $\beta$, based on our results and accounting for energy conservation, is shown in a partial dependency plot (Fig. 13). Since stress anisotropy cannot be considered independent from the Froude number, we define $\mu$, which gives $\kappa _1$ as a function of ${\rm Fr}$. The corresponding fixed values of the partial dependency plot refer to the respective mean values, $\langle \kappa _1\rangle = 2.19$ or $\langle \delta \rangle = 1.23$, of all conducted impact related replicates. Thus we define $\mu _e$, related to the energy conservation approach (Eq. 9) with:

20 $$\begin{aligned} \mu _e=2.19+\frac{{\rm Fr}^2}{2} \end{aligned}$$

Comparing the influence of $\mu _e$ and $\delta$ for the prediction of $\beta$, once based on equation (Eq. 10) and once based on equation (Eq. 9), it is found that for the same Froude condition, indicated as an example with ${\rm Fr}=1$ in Fig. 13, higher run-up coefficients are estimated when accounting for $\kappa _1$ and $\delta$. As indicated by the circle markings at ${\rm Fr}=1$ in Fig. 13, it further shows that for the model based on energy conservation, stress anisotropy has a greater influence on the estimation of the possible run-up coefficients than density variations due to impact. This is also evident in Fig. 11, wherein the lower Froude ranges higher $\kappa _1$ values are reflected in significantly higher $\beta$ values.

The approach of mass and momentum conservation, represented by the MJ model, does not ensure the conservation of mechanical energy, and leads therefore most likely to an underestimation of run-up coefficients at higher Froude numbers. As proposed by [5], a potential transition between mass and momentum and energy conservation approaches can be assumed near a Froude number of three. In fact, for ${\rm Fr}=3$, Eq. (13), based on the mass and momentum balance of a homogeneous fluid, approximates Eq. (10)—which in turn is based on energy conservation.

**Fig. 14:**
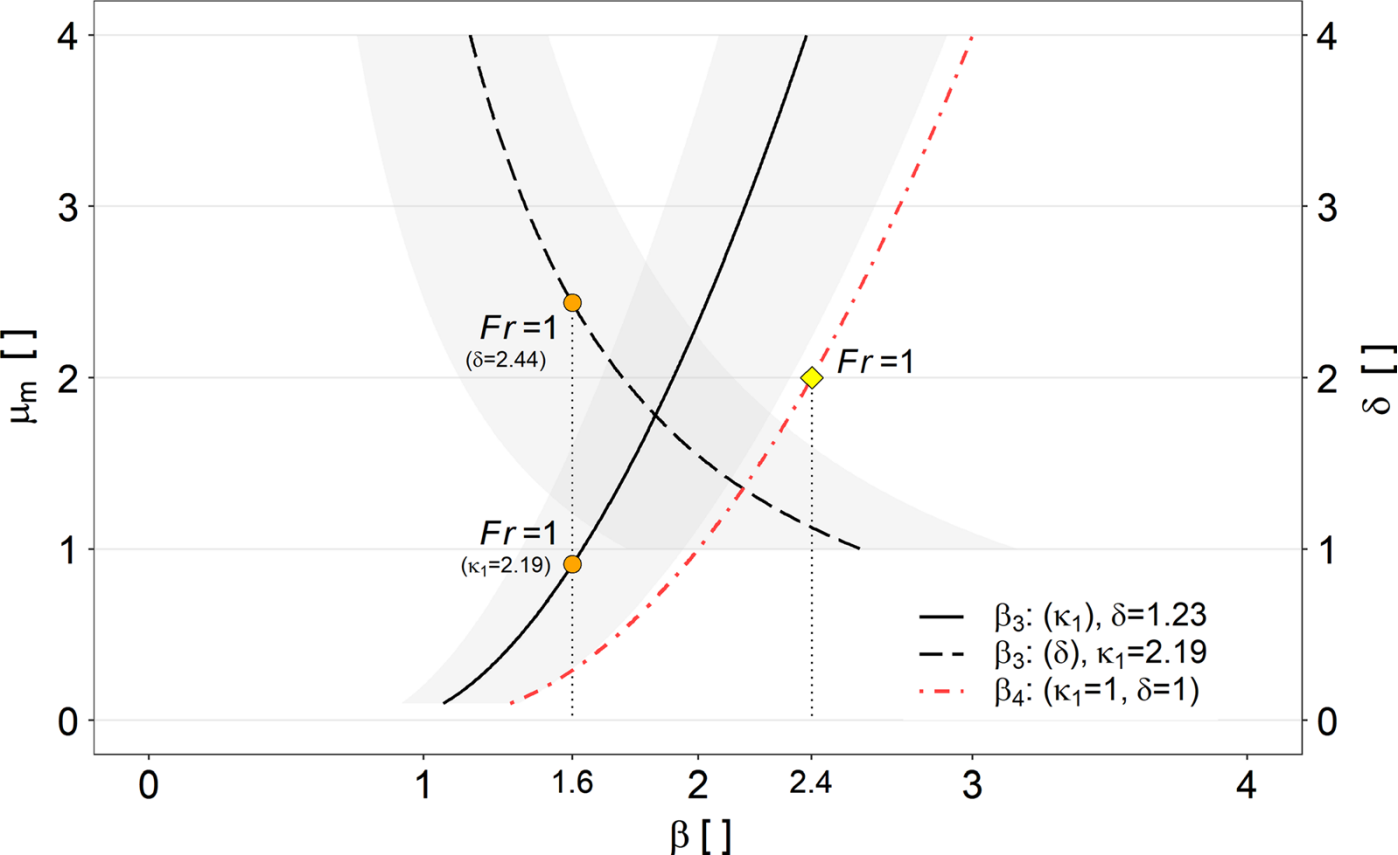
Partial dependency plot of $\mu _m$ and $\delta$ to calculate the run-up coefficient ($\beta _3$) based on Eq. (12). Here, $\mu _m$ is given as a function of $\kappa _1$ and ${\rm Fr}$ (Eq. 21). The fixed values correspond to the respective mean values, $\langle \kappa \rangle =2.19$ or $\langle \delta \rangle =1.23$ of all conducted impact related replicates. The range of uncertainty is based on one standard deviation from the fixed mean values. Also shown is the influence of $\mu _m$ on the calculation of the run-up coefficient $\beta _4$, when based on Eq. (13b), with $\delta =1$ and $\kappa =1$. The markers correspond to $\mu _e$ respectively $\kappa _1$ or $\delta$ values valid at ${\rm Fr}=1$, either estimated based on Eqs. (12) or (13b), respectively

**Fig. 15:**
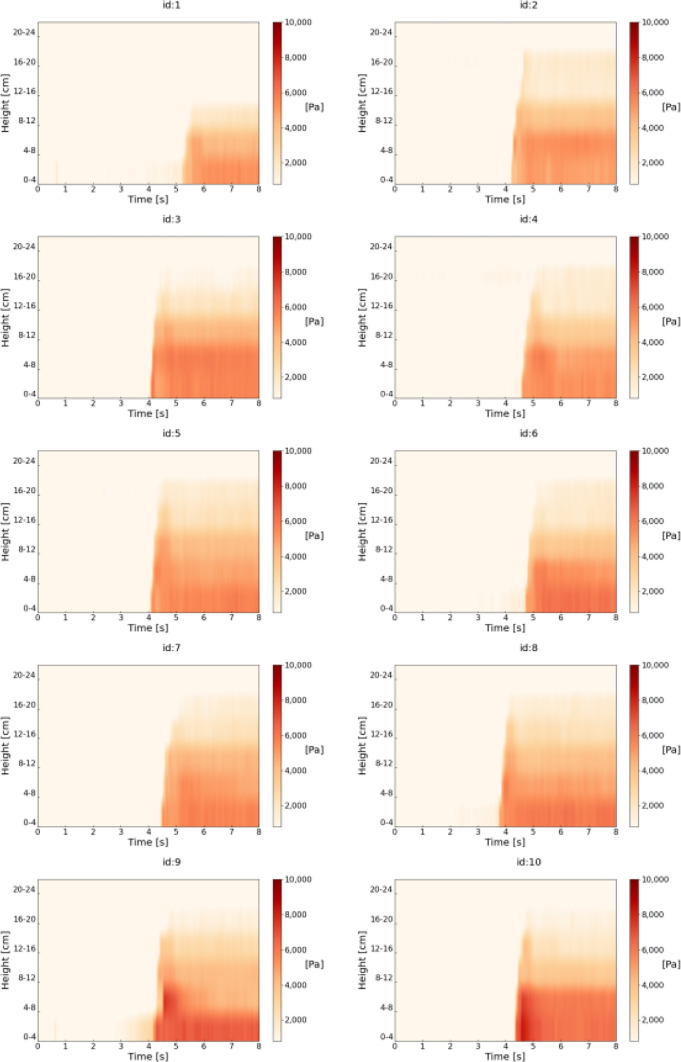
Progression of measured impact pressures over time and height of the impact measurement device for replicates based on the coarse mixture

**Fig. 16:**
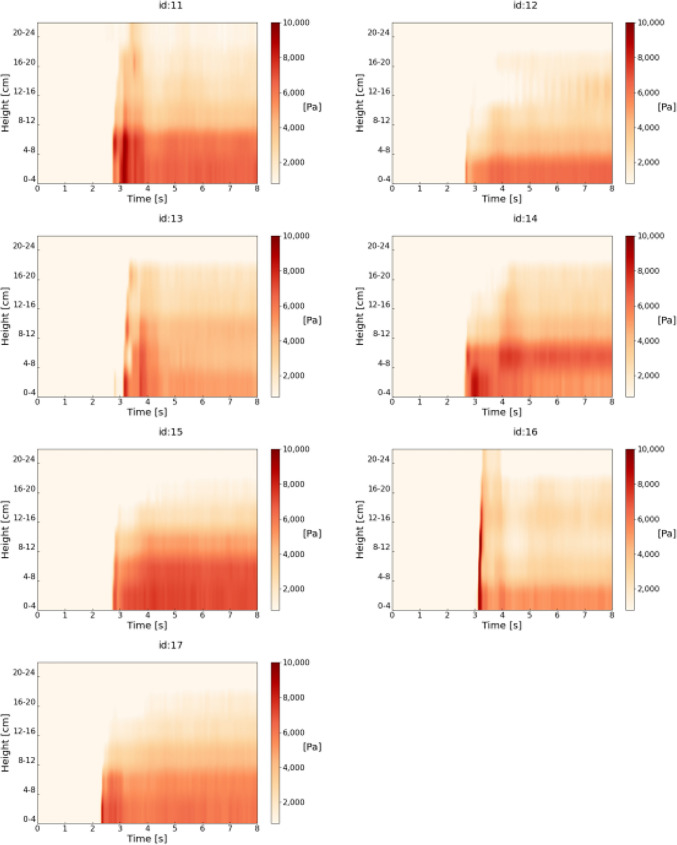
Progression of measured impact pressures over time and height of the impact measurement device for replicates based on the muddy mixture

The dependency of density ratio ($\delta$) and bulk stress anisotropy ($\kappa _1$), for estimating the run-up coefficient ($\beta _3$) based on Eq. (12), is shown in Fig. 14. Due to the correlation of $\kappa _1$ with the Froude number, we introduce $\mu _m$ related to the mass and momentum conservation approach with:

21 $$\begin{aligned} \mu _m=\frac{2{\rm Fr}^2}{2.19} \end{aligned}$$

The influence on $\beta _3$, shown in Fig. 14, applies to both parameters, $\mu _m$ and $\delta$. In contrast to the energy conservation approach, run-up estimation based on mass and momentum conservation shows for the same Froude conditions lower run-up coefficients when accounting for stress anisotropy and density variations (Fig. 14). For the estimation of the run-up coefficient $\beta _3$, based on the momentum jump model which considers $\kappa =1$ as well as $\delta =1$ (12), our results further indicate that the density variation at impact, in contrast to the energy conservation approach, is the more decisive variable (c.f. circle markings in Fig. 14).

## Conclusions

The expected peak impact pressure of a granular debris flow can be calculated based on the hydrostatic pressure head, which is directly related to the run-up height as well as stress anisotropy and bulk flow density at impact. The considered impact model applies to debris flows at low Froude ranges whose bulk flow resistance is dominated by frictional or collisional grain interactions. Here, no or only little energy of the impacted mass is dissipated, assuming that the entire mechanical energy of the inflow is converted into potential energy upon impact.

Based on impact tests of experimental debris flows with a small-scale physical model, this study provides information on measured stress anisotropy coefficients, bulk flow densities as well as run-up, back calculated from the impact measurements. These results are applied to, and compared with existing run-up models, which are either based on mass and momentum or energy conservation and in their most explicit form considering stress anisotropy and density variation of the flow at impact. Our results show that for the prototypical debris flows investigated in this study, the performance of run-up models based on energy conservation in low Froude ranges depends essentially on stress anisotropy and less on density variations at impact. This is in plausible contrast to the performance of run-up models based on mass and momentum balance. Here, it seems that in the low Froude ranges the density variations at impact are more decisive. However, our results indicate that especially in these low Froude ranges, knowledge of anisotropic stress ratios as well as density variations at impact, reduce uncertainties for the determination of maximum debris-flow impact pressures.

The considered impact model in this study offers the possibility to calculate the maximum impact pressures of granular debris flows based on impact dynamics and run-up height. It theoretically enables future studies to back-calculate potential impact forces based on post-event field investigations. In the specific case of estimating Froude depending run-up heights, such field investigations can be based on geological deposits on banks [45] or on flow traces on trees, rocks or walls. However, further controlled experiments are needed and planned with the experimental setup presented here, for the Froude range $2.5<{\rm Fr}<5.5$—where both theoretical run-up model approaches overlap and where we expect a shift in significance from hydrostatic to dynamic pressure components for the determination of the maximum debris-flow impact pressure [13].

## Data Availability

The data on which the results—presented in this study—are based, can be found in the appendix. All measured data for each replicate are available from the corresponding author upon request (flow heights, horizontal forces, normal forces, pore water pressures).

## Appendix A: Notation

*The following symbols are used in this paper:*

**Table:**

$A_S$	Sensor area of the normal force measurement device (${{{\rm m}}^{2}}$)
$C_V$	Solid concentration by volume (–)
*d*	Characteristic grain diameter (m)
${\rm Fr}$	Froude number (–)
$F_N$	Normal force (N)
$h_0$	Flow height of the incoming flow (m)
$h_1$	Maximum run-up height at impact (m)
${\rm LR}$	Liquefaction ratio (–)
$N_B$	Bagnold number (–)
$N_S$	Savage number (–)
$p_f$	Pore fluid pressure (Pa)
$p_{{\rm peak}}$	Peak impact pressure predicted by theoretical model (Pa)
$p_{\max }$	Maximum measured impact pressure (Pa)
*S*	Ratio between predicted and measured impact pressures (–)
$v_0$	Flow velocity^a^ (ms^-1^)
$v_1$	Flow velocity at the moment of the maximum run-up height (ms^-1^)
$\beta$	Run-up coefficient (–)
$\beta ^*$	Back-calculated run-up coefficient (–)
$\delta$	Bulk density ratio (–)
$\eta _f$	Fluid viscosity (Pa s)
$\kappa _0$	Normal stress anisotropy coefficient of the incoming flow (–)
$\kappa _1$	Normal stress anisotropy coefficient at impact (–)
$\rho _0$	Bulk density of the incoming flow (kg m^-3^)
$\rho _1$	Bulk density of the impacted flow (kg m^-3^)
$\rho _b$	Bulk density at rest (kg m^-3^)
$\rho _s$	density of the solid particles (kg m^-3^)
$\rho _f$	Fluid density (kg m^-3^)
$\sigma _{{\rm tot}}$	Total normal stress (Pa)
$\sigma _{{\rm eff}}$	Effective normal stress (Pa)
$\sigma _{{\rm stat}}$	Hydrostatic stress (Pa)
$\theta$	Slope angle (flume slope = 20°)
${\dot{\gamma }}$	shear rate (s^-1^)

^a^Corresponds to the maximum front velocity for this study

## Appendix B: Compiled data of the individual replicates with impact measurement device

Table 2 lists all values for all replicates where impact was measured. The flow velocities ($v_0$), flow heights ($h_0$), maximum impact pressures ($p_{\max }$), total normal stresses ($\sigma _{{\rm tot}}$), pore fluid pressures ($p_{f}$) before impact, as well as bulk densities of the impacted flow ($\rho _1$) have all been directly measured for each experiment. Froude numbers (${\rm Fr}$), liquefaction ratios (${\rm LR}$), anisotropy coefficients ($\kappa$) and bulk density ratios ($\delta$) have been directly derived. The run-up coefficients $\beta ^*$ are back-calculated, based on Eq. (19). All units and definitions of parameters and variables are given in the notation (Appendix A).

**Table 2:** Compiled values of all experiments with impact measurement device. Units are given in Appendix (A)

$\#$	Mixture	$v_0$	$h_0$	${\rm Fr}$	$p_{\max }$	$\sigma _{{\rm tot}}$	$p_{f}$	${\rm LR}$	$\kappa _1$	$\rho _1$	$\delta$	$\beta ^*$
1	Coarse	0.86	0.11	0.84	5842	1803	1899	0.69	3.24	1759	0.98	1.00
2	Coarse	1.41	0.10	1.47	5576	2092	2309	0.60	2.67	1837	1.03	1.23
3	Coarse	1.45	0.09	1.57	6079	4165	2387	0.54	1.46	4120	2.30	1.20
4	Coarse	1.21	0.11	1.19	6904	2639	2388	0.59	2.62	1909	1.07	1.32
5	Coarse	1.30	0.10	1.34	7156	3727	2472	0.57	1.92	1994	1.11	1.99
6	coarse	1.50	0.11	1.47	8304	3164	2416	0.59	2.62	1899	1.06	1.58
7	Coarse	1.38	0.11	1.38	8013	3674	2392	0.58	2.18	2172	1.21	1.71
8	Coarse	1.43	0.12	1.34	8336	3435	2465	0.57	2.43	1987	1.11	1.51
9	Coarse	1.44	0.11	1.40	11,145	2234	2438	0.72	4.99	1442	0.84	1.47
10	Coarse	1.39	0.12	1.32	8229	3315	2415	0.54	2.48	2022	1.18	1.48
Mean		1.34	0.11	1.33	7,558	3,025	2,358	0.60	2.66	2,114	1.19	1.45
Standard deviation		$\pm 0.18$	$\pm 0.01$	$\pm 0.19$	$\pm 1560$	$\pm 750$	$\pm 159$	$\pm 0.06$	$\pm 0.90$	$\pm 694$	$\pm 0.38$	$\pm 0.27$
11	Muddy	2.23	0.09	2.41	11,870	3112	3849	0.79	1.35	1822	1.06	5.60
12	Muddy	1.91	0.08	2.21	6576	2512	3204	0.71	1.79	2091	1.22	2.33
13	Muddy	1.96	0.09	2.10	11,098	1848	3345	0.88	1.73	1598	0.93	4.61
14	Muddy	1.95	0.08	2.30	8534	3431	3306	0.66	1.20	2456	1.43	4.02
15	Muddy	2.03	0.08	2.31	7550	3927	3074	0.75	1.92	1966	1.14	2.60
16	Muddy	1.87	0.13	1.71	13,285	5178	3597	0.69	1.35	3116	1.81	2.64
17	Muddy	1.89	0.09	2.13	7782	3324	3554	0.63	1.35	2379	1.38	3.06
Mean		1.98	0.09	2.17	9528	3333	3418	0.73	1.53	2204	1.28	3.55
Standard deviation		$\pm 0.11$	$\pm 0.02$	$\pm 0.21$	$\pm 2352$	$\pm 978$	$\pm 245$	$\pm 0.08$	$\pm 0.26$	$\pm 464$	$\pm 0.27$	$\pm 1.13$

## Appendix C: Compiled data of the individual replicates without impact measurement device

Table 3 lists all the values for all replicates without impact measurements. The flow velocities ($v_0$), flow heights ($h_0$), total normal stresses ($\sigma _{{\rm tot}}$), pore fluid pressures ($p_{f}$), as well as inflow bulk densities ($\rho _0$) have all been directly measured for each experiment. Froude numbers (${\rm Fr}$) and liquefaction ratios (${\rm LR}$) have been directly derived. All units and definitions of parameters and variables are given in the notation (Appendix A).

**Table 3:** Compiled values of all experiments without impact measurement device. Units are given in appendix (A)

$\#$	Mixture	$v_0$	$h_0$	${\rm Fr}$	$\sigma _{{\rm tot}}$	$p_{f}$	${\rm LR}$	$\rho _0$
18	Coarse	1.02	0.105	1.01	1766	1025	0.58	1775
19	Coarse	1.30	0.107	1.27	1688	1010	0.60	1779
20	Coarse	1.01	0.098	1.03	1590	1110	0.69	1819
Mean		1.11	0.10	1.10	1681	1048	0.63	1791
Standard deviation		$\pm 0.14$	$\pm 0.00$	$\pm 0.12$	$\pm 72$	$\pm 44$	$\pm 0.05$	$\pm 20$
21	Muddy	1.71	0.11	1.62	1828	1401	0.77	1,740
22	Muddy	1.68	0.12	1.53	1578	1184	0.75	1,859
23	Muddy	1.62	0.12	1.51	1811	1414	0.78	1,561
Mean		1.67	0.12	1.55	1739	1333	0.77	1,720
Standard deviation		$\pm 0.04$	$\pm 0.00$	$\pm 0.05$	$\pm 114$	$\pm 105$	$\pm 0.01$	$\pm 122$

## Appendix D: Impact pressures over time and height

See Figs. 15 and 16.
